# PD-L1 tumour expression is predictive of pazopanib response in soft tissue sarcoma

**DOI:** 10.1186/s12885-021-08069-z

**Published:** 2021-03-31

**Authors:** Sang Kyum Kim, Jee Hung Kim, Seung Hyun Kim, Young Han Lee, Jung Woo Han, Wooyeol Baek, Ha Young Woo, Min Kyung Jeon, Hyo Song Kim

**Affiliations:** 1 Department of Pathology, Yonsei University College of Medicine, Seoul, South Korea; 2 Division of Medical Oncology, Department of Internal Medicine, Gangnam Severance Hospital, Yonsei University College of Medicine, Seoul, South Korea; 3 Department of Orthopedic Surgery, Yonsei University College of Medicine, Seoul, South Korea; 4 Department of Radiology, Yonsei University College of Medicine, Seoul, South Korea; 5 Division of Pediatric Hemato-Oncology, Department of Pediatrics, Yonsei University College of Medicine, Seoul, South Korea; 6 Department of Plastic Surgery, Yonsei University College of Medicine, Seoul, South Korea; 7 Division of Medical Oncology, Department of Internal Medicine, Yonsei Cancer Center, Yonsei University College of Medicine, Seoul, South Korea

**Keywords:** Soft tissue sarcoma, Pazopanib, PD-L1, Biomarker

## Abstract

**Background:**

Pazopanib, a multitargeted tyrosine kinase inhibitor, is recommended as the standard treatment for refractory soft tissue sarcoma (STS). However, there are comparatively few molecular determinants for predicting pazopanib efficacy. Based on correlative studies regarding the predictive impact of PD-L1, we investigated the clinical relevance of PD-L1 expression and evaluated its value for predicting pazopanib efficacy.

**Methods:**

Tumour tissues from patients with advanced STS who went on to receive pazopanib were assessed for PD-L1 expression. Immunohistochemistry was performed using an anti-PD-L1 antibody, and the PD-L1 tumour proportion score (TPS) was calculated as the percentage of at least 100 viable cells with positive expression, defined as TPS ≥ 1%.

**Results:**

Among the 67 patients, 8 (11.9%) achieved partial response and a median progression-free survival (PFS) of 4.8 months (95% CI 3.8–5.7). PD-L1 expression in tumour cells was detected in 13 (19.4%) cases and the TPS scores ranged from 1 to 100%, as follows: 0 (*n* = 54, 80.6%), 1–9% (*n* = 3, 4.5%), 10–49% (*n* = 9, 13.4%), and ≥ 50% (*n* = 1, 1.5%). PD-L1 positive tumours exhibited a poorer response to pazopanib treatment than the PD-L1 negative tumours (0% vs 14.8%, *P* = 0.07). PD-L1-positive tumours had significantly shorter PFS than the PD-L1-negative tumours (median PFS 2.8 vs 5.1 months, *P* = 0.003), and PD-L1 positivity was an independent predictor of poor response to pazopanib treatment (HR 2.77, 95% CI; 1.45–5.56, *P* = 0.006).

**Conclusion:**

We identified that PD-L1 expression can help predict the clinical outcome of patients with advanced STS treated with pazopanib. Based on our study, stratification should be actively considered in order to identify patients who will benefit from pazopanib or further therapeutic strategies for future clinical trials.

**Supplementary Information:**

The online version contains supplementary material available at 10.1186/s12885-021-08069-z.

## Background

Soft tissue sarcomas (STSs) comprise a rare and heterogeneous group of tumours that originate from mesenchymal cells and account for 1% of all adult malignancies [1]. Although the primary treatment is usually surgery and/or radiotherapy, up to 40% of the patients experience tumour recurrence, and the survival rate is poor, with a median survival overall of 12 months [2].

For patients with advanced STS, palliative chemotherapy generally takes the form of doxorubicin- or ifosfamide-based regimens [2, 3]. If first-line treatment fails, trabectedine, dacarbazine and gemcitabine, and/or docetaxel, have been approved as salvage treatments. Pazopanib is a multitargeted tyrosine kinase inhibitor that shows activity against vascular endothelial growth factor, platelet-derived growth factor, fibroblast growth factor receptors, and c-kit. In a stratified phase II trial for advanced STS [4], pazopanib displayed anti-cancer activity against leiomyosarcoma and synovial sarcoma, but not liposarcoma. In a subsequent phase III Pazopanib for Metastatic Soft-Tissue Sarcoma (PALETTE) trial designed for investigating the effect of pazopanib on non-adipocytic STS, it was observed that pazopanib treatment resulted in an improved progression-free survival (3 months) compared to that observed in response to the placebo [5]. While pazopanib is currently recommended as a standard treatment, patient response to this drug is still modest and the improvement of overall survival is not significant. We hypothesise that identifying molecular predictors to select patients who might benefit from pazopanib treatment might improve the efficacy of this drug. While previous studies have reported a number of clinical parameters as predictors of pazopanib efficacy, including circulating angiogenic factors and neutrophil to lymphocyte ratios, the application of these parameters has been limited thus far [6, 7].

Based on our understanding of the mechanisms by which tumours evade the immune response, immune checkpoint inhibitor-based therapies (e.g.: pembrolizumab monotherapy, combinatorial therapy with nivolumab and ipilimumab, a cytotoxic T-lymphocyte–associated antigen 4 (CTLA-4) inhibitor) have been developed which show an effect on advanced STS [8, 9]. Programmed death-ligand 1 (PD-L1) is aberrantly expressed in several subtypes of STS and has been associated with poor prognosis and adverse features [10, 11]. Recently, a correlation between PD-L1 expression and clinical outcome was reported in renal cell carcinoma (RCC). Choueiri et al. showed that patients with higher PD-L1 expression had worse overall survival when treated with pazopanib or sunitinib [12]. However, the predictive impact of PD-L1 in STS remains unclear, and further research is warranted to provide more detailed therapeutic guidance regarding the treatment of this disease.

In this study, we investigated PD-L1 expression in STS tissues and evaluated the clinical relevance of its expression in different STS subtypes. In addition, we also evaluated the correlation between the expression of PD-L1 and treatment outcomes of patients who received pazopanib, for facilitating the identification of a molecular biomarker that could aid the design of effective STS treatments.

## Methods

### Patient and study procedure

We retrospectively selected 91 patients who were pathologically diagnosed with STS, from September 2013 to December 2019, at Yonsei Cancer Center, Yonsei University College of Medicine. Among these patients, 24 were excluded due to the lack of available specimens (*n* = 13) or insufficient tumour content (*n* = 11); 67 patients were finally included in this study. To select the most representative formalin-fixed paraffin-embedded (FFPE) tissues for immunohistochemistry, samples were mounted on slides, stained with haematoxylin and eosin, and reviewed by two pathologists (SKK and HYW). These tissue used for analysis were before pazopanib treatment.

Clinical information, including age, sex, etiology, Eastern Cooperative Oncology Group (ECOG) performance status, French Federation of Cancer Centers Sarcoma Group (FNCLCC) system score, staging and previous treatment data, were extracted from the hospital records. The study was reviewed and approved by the Institutional Review Board of the Yonsei Cancer Center (IRB. 4–2017-1023) in accordance with the Declaration of Helsinki and the Guidelines for Good Clinical Practice.

### Immunohistochemistry and evaluation of PD-L1 expression and tumour infiltrating lymphocytes (TIL) score

In patients treated with pazopanib in combination with anti-PD-L1 blockade, PD-L1 expression was analysed using an anti-PD-L1 antibody (clone SP263, Ventana). From each block, 5 μm sections were cut and stained using the anti-PD-L1 antibody on an automated staining platform (Benchmark ULTRA; Ventana). An OptiView DAB IHC Detection Kit and an OptiView Amplification Kit (both Ventana) were used according to the manufacturer’s recommendations for the visualisation of PD-L1 protein. The PD-L1 tumour proportion score (TPS) was expressed as the percentage of at least 100 viable cells exhibiting complete or partial membrane staining and a three-tiered system was then applied using the following thresholds: < 1%, 1–49% and ≥ 50%. Positive expression was defined as TPS ≥ 1%. Macrophages were used as an internal control in order to validate the adequacy of the PD-L1 staining.

The percentage of intratumoural and stromal tumour infiltrating lymphocytes (TILs) was evaluated using the criteria described and published by the International Immuno-Oncology Biomarker Working Group [13]. Briefly, all mononuclear cells, excluding neutrophils, were scored, and the average number of TILs in the tumour area was assessed as a continuous variable. TIL scores were classified into the following three groups: negative (< 1%), low (1–9%), intermediate (10–59%), and high (≥ 60%), by adopting the definition published by Ogiya et al. [14]

### Statistical analysis

Progression-free survival (PFS) was defined as the time from the start of pazopanib treatment until the date of disease progression or death resulting from any cause. Overall survival (OS) was measured from the start of pazopanib treatment with advanced STS to the date of death due to any cause. Survival difference was analysed using the Kaplan–Meier method and was assessed using the log-rank test. Overall response rate (ORR) was calculated as the percentage of patients experiencing a confirmed complete response (CR) or partial response (PR), as per the Response Evaluation Criteria in Solid Tumors (RECIST) 1.1 guidelines. The associations between clinical features and each of the dichotomised groups were analysed using the chi-square test or Fisher’s exact test. All *P*-values were two-sided and *p* < 0.05 was considered significant. Statistical analysis was conducted using SPSS (version 21.0; SPSS, Inc., Chicago, IL) and GraphPad Prism version 5 (Graph Pad Software Inc., San Diego, CA, USA.

## Results

### Clinicopathological characteristics of sarcoma patients

FFPE primary tumour specimens from a total of 67 patients with unresectable or metastatic STS were analysed for PD-L1 expression using immunohistochemistry. Fifty-two cases (77.6%) were made from surgical specimens and 15 (22.4%) from biopsy samples. And 58 cases (86.6%) were from the primary tumor site and 9 (13.4%) from metastasis sites. Twelve cases (17.9%) were tissue samples obtained after chemotherapy, of which 7 cases were after the first chemotherapy and 5 cases were after the second chemotherapy or more. The most commonly observed histologies were leiomyosarcoma (*n* = 18), undifferentiated pleomorphic sarcoma (UPS; *n* = 13), angiosarcoma (*n* = 8), and synovial sarcoma (*n* = 6). The majority of the tumours were high grade (FNCLCC grade 2 or 3; 91.0%) and 61.2% of them arose in the abdomen or thorax. All patients had received at least one previous regimen of chemotherapy, mainly doxorubicin or gemcitabine plus docetaxel based (Table 1).

**Table 1:** **Patient characteristics.**

Variables	Total	PD-L1 (−)	PD-L1 (+)	***P*** value
		**54 (80.6%)**	**13 (19.4%)**	
**Age (Median)**	48	51 (38–73)	45 (22–72)	
**Sex**				0.82
Male	38	31 (81.6%)	7 (18.4%)	
Female	29	23 (79.3%)	6 (20.7%)	
**ECOG**				0.99
0	3	3 (100%)	0 (0.0%)	
1	36	28 (77.8%)	8 (22.2%)	
2	26	21 (80.8%)	5 (19.2%)	
Not available	2	2 (100%)	0 (0.0%)	
**Histologic variant**				0.22
Leiomyosarcoma	18	15 (83.3%)	3 (16.7%)	
Undifferentiated pleomorphic sarcoma	13	9 (69.2%)	4 (30.8%)	
Angiosarcoma	8	5 (62.5%)	3 (37.5%)	
Synovial sarcoma	6	6 (100%)	0 (0.0%)	
Myofibroblastic sarcoma	6	5 (83.3%)	1 (16.7%)	
MPNST	5	3 (60%)	2 (40%)	
Etc*	11	11 (100%)	0 (0.0%)	
**Primary site**				0.23
Abdomen/pelvis	23	20 (87.0%)	3 (13.0%)	
Extremity	22	19 (86.4%)	3 (13.6%)	
Thorax	18	13 (72.2%)	5 (27.8%)	
Head/neck	4	2 (50%)	2 (50%)	
**FNCLCC grade**				0.36
I	6	6 (100%)	0 (0.0%)	
II	33	27 (81.8%)	6 (19.2%)	
III	28	21 (75%)	7 (25%)	
**Number of previous chemotherapy**				0.83
1	48	38 (79.2%)	10 (20.8%)	
2	18	15 (83.3%)	3 (16.7%)	
3	1	1 (100%)	0 (0.0%)	
**Type of previous chemotherapy received**				NA
Doxorubicin monotherapy	9	7 (77.8%)	2 (22.2%)	
Ifosfamide monotherapy	3	2 (66.7%)	1 (33.3%)	
Paclitaxel	4	3 (75.0%)	1 (25.0%)	
Doxorubicin combination	33	28 (84.8%)	5 (15.2%)	
Ifosfamide combination	4	3 (66.7%)	1 (33.3%)	
Cyclophosphamide based	14	11 (78.6%)	3 (21.4%)	
Gemcitabine/docetaxel	14	12 (85.7%)	2 (14.3%)	

* 8th edition of the American Joint Committee on Cancer guideline of tumor, node, and metastasis (TNM) classification

*Etc: ASPS (alveolar soft part sarcoma, n = 3), epithelioid sarcoma (n = 3), DSRCT (desmoplastic small round cell tumors, n = 1), osteosarcoma (n = 1), liposarcoma (n = 1), rhabdomyosarcoma (n = 1), and PECOMA (perivascular epithelioid cell tumors, n = 1)

Abbreviation: Eastern Cooperative Oncology Group (ECOG), malignant peripheral nerve sheath tumor (MPNST), Fédération Nationale des Centres de Lutte Contre le Cancer (FNCLCC)

### Correlation between PD-L1 expression and the TIL score

**Fig. 1:**
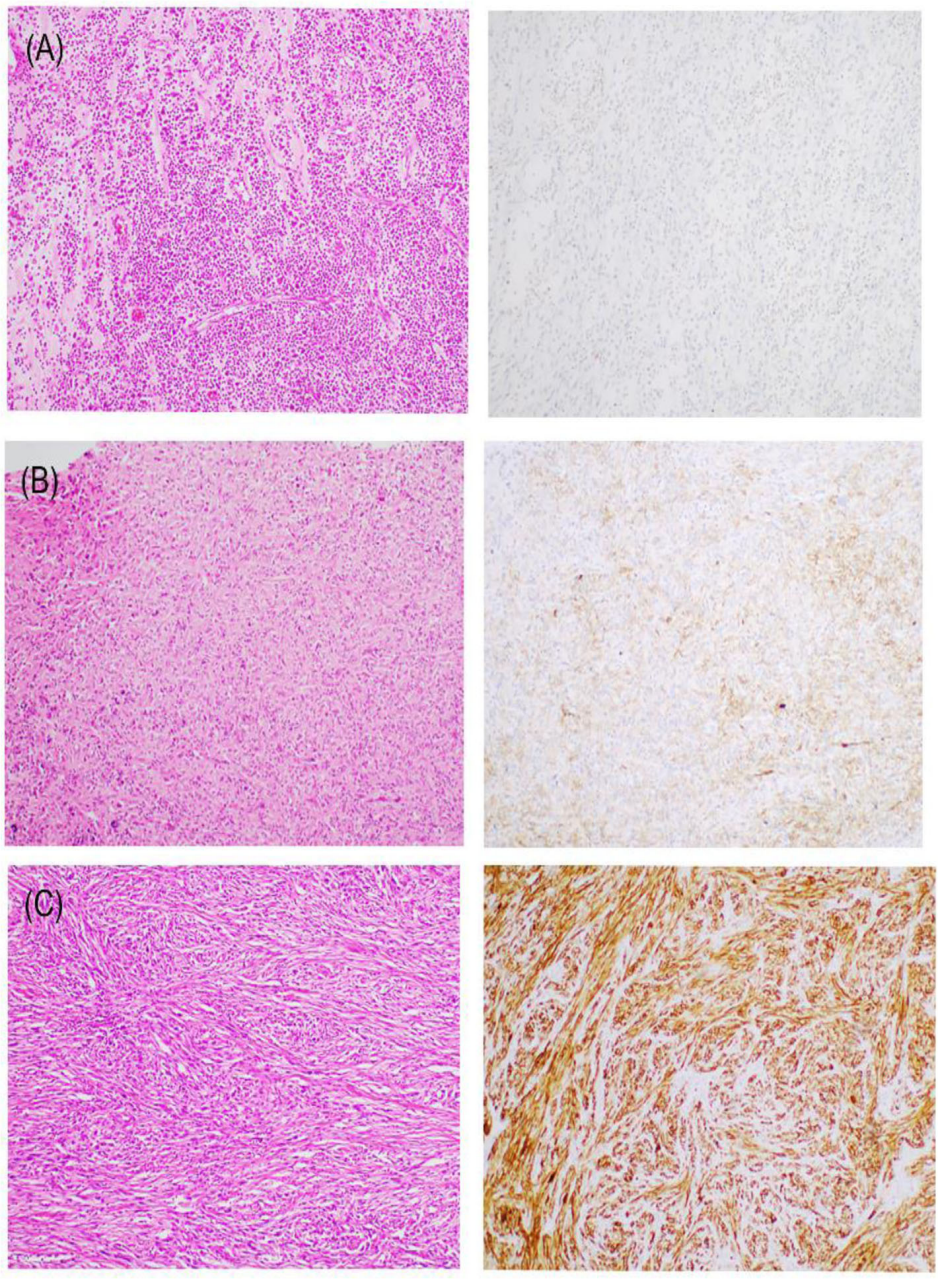
Immunohistochemistry for PD-L1 expression in STS. Representative images of PD-L1-negative (**a**, TPS 0%, × 100) and -positive cases (**b**, TPS 40%, × 100) (**c**, TPS 100%, × 100). *Abbreviation: Programmed death-ligand 1(PD-L1), soft tissue sarcoma (STS), tumour proportion score (TPS)*

Overall, PD-L1 expression in the tumour cells was detected in 13 (19.4%) of the 67 patients, with TPS scores ranging from 1 to 100% positive staining in the following manner: 0 (*n* = 54, 80.6%), 1–9% (*n* = 3, 4.5%), 10–49% (*n* = 9, 13.4%), and ≥ 50% (*n* = 1, 1.5%) positive staining. Representative images are provided in Fig. 1. PD-L1-positive (+) cases were common in UPS, angiosarcoma, malignant peripheral nerve sheath tumour (MPNST), and leiomyosarcoma but none of the synovial sarcoma tumours exhibited PD-L1 expression. PD-L1 (+) patients tended to present a higher grade and head/neck primary tumour.

The inflammatory response, as represented by the presence of TILs, was simultaneously evaluated in the following manner: negative (*n* = 39, 58.2%), low TIL (*n* = 16, 23.9%), intermediate TIL (*n* = 11, 16.4%), and high TIL (n = 1, 1.5%) sarcoma. There was a possible correlation between PD-L1-positive staining and TILs, and the percentage of TILs showed a tendency to increase according to the degree of PD-L1-positive staining (*P* = 0.09, Table 2). No significant difference was detected when the TIL score was evaluated with respect to other clinical characteristics (age, sex, ECOG performance, histologic subtype, and primary tumour site).

**Table 2:** Correlation between PD-L1 expression and TIL

		PD-L1 (%)				Total
		**0**	**1–9**	**10–49**	**≥50**	**P = 0.09**
TIL	**Negative (0)**	34 (62.9%)	1 (33.3%)	4 (44.4%)	0	39 (58.2%)
	**Low (1–9)**	13 (24.1%)	2 (66.7%)	1 (11.1%)	0	16 (23.9%)
	**Intermediate (10–59)**	6 (11.1%)	0	4 (44.4%)	1 (100%)	11 (16.4%)
	**High (≥60)**	1 (1.9%)	0	0	0	1 (1.5%)
		54 (80.6%)	3 (4.5%)	9 (13.4%)	1 (1.5%)	

Abbreviation: Programmed death-ligand 1(PD-L1), tumour infiltrating lymphocytes (TILs)

### Correlation between PD-L1 expression with pazopanib efficacy

**Fig. 2:**
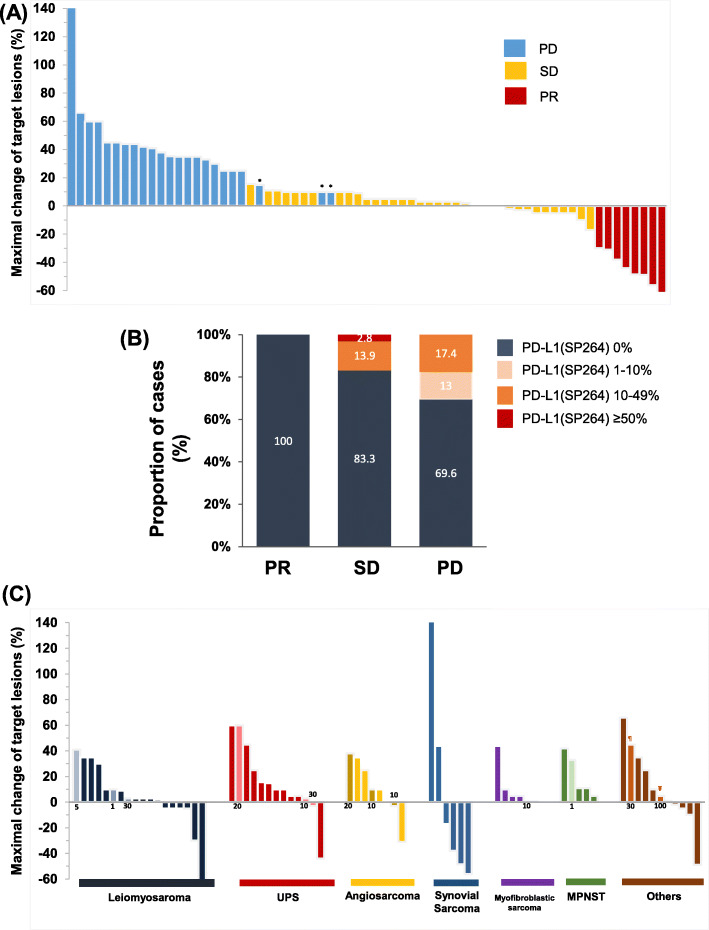
Maximum response to pazopanib in patients with STS. (**a**) The waterfall plot represents percentage of maximum tumour reduction in response to treatment, assessed according to RECIST 1.1 criteria. * indicates newly developed lesions per RECIST 1.1. (**b**) Prevalence of PD-L1 expression in responders (PR) and non-responders (SD, PD), assessed according to RECIST 1.1 criteria. (**c**) Maximum tumour reduction according to histological subtypes and PD-L1 status. PD-L1 TPS score (≥1%) has been plotted on the X-axis and PD-L1 positive marked in light color bars. Indicates rhabdomyosarcoma and ¥ indicates ASPS (Alveolar soft part sarcoma). Abbreviation: Soft tissue sarcoma (STS), Response Evaluation Criteria in Solid Tumors (RECIST) 1.1, Programmed death-ligand 1(PD-L1), tumour proportion score (TPS), PR (partial response), SD (stable disease), PD (progression disease). *This figure was generated with Microsoft Excel

Of the 67 patients evaluated, 8 achieved a PR, 36 had stable disease, and 23 exhibited progressive disease phenotypes, resulting in an ORR of 11.9% (Fig. 2a). Responses were first detected at a median of 2.3 months (range 1.8–8.2) after treatment initiation and lasted for a median of 6.5 months (range 2.1–19.6). Responder who showed PR had higher PD-L1 negativity than non-responder who showed SD and progression disease (PD) (100% versus 83.3% versus 69.6%, Fig. 2b). The ORRs of the different histological subtypes were as follows; 2 PRs (11.1%) among 18 leiomyosarcomas, 1 (7.7%) in 13 UPS, 1 (12.5%) of 8 angiosarcomas, and 1 (33.3%) of 3 epithelioid sarcomas. In synovial sarcoma, 3 PRs (50%) were detected, of which 2 were biphasic and 1 was monophasic (Fig. 2c). Brief case descriptions of PD-L1 (+) expression are supplied in Supplementary Table 1.

**Fig. 3:**
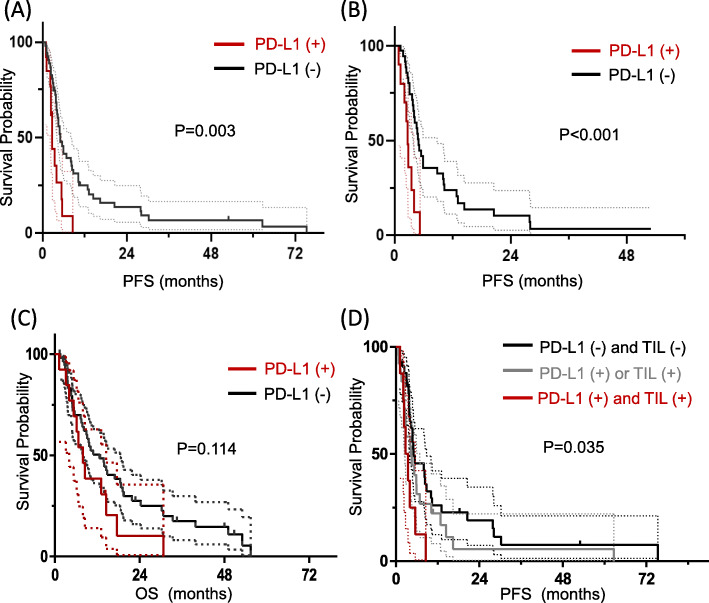
Survival analyses based on PD-L1 expression. Kaplan–Meier survival curves for PFS for all patients (**a**) and those with second-line treatment (**b**). Kaplan–Meier survival curves for OS (**c**). PFS difference in response to the combined expression of PD-L1 and TILs (**d**). *Abbreviation: Programmed death-ligand 1(PD-L1), progression-free survival (PFS), overall survival (OS), tumour infiltrating lymphocytes (TILs)*. *This figure was generated with GraphPad Prism version 5 (Graph Pad Software Inc., San Diego, CA, USA)

Fifty-nine patients (88.1%) exhibited progressive events and the median PFS was 4.8 months (95% CI 3.8–5.7). Patients with PD-L1 (+) expression had significantly shorter PFS than PD-L1 (−) patients (median PFS 2.8 vs 5.1 months, *P* = 0.003, Fig. 3a). This trend was consistent for second-line–treated patients (Fig. 3b, median 2.8 vs 5.0 months, *P* < 0.001). The median OS was 10.1 months (95% CI 5.42–14.78), and PD-L1-negative patients had a slightly better OS than PD-L1-positive patients, although this was not significant (12.6 vs 7.9 months, *P* = 0.11, Fig. 3c).

In multivariate Cox regression analyses, only PD-L1 (+) expression was identified to be an independent factor for determining poor PFS upon pazopanib treatment (HR 2.77, 95% CI; 1.45–5.56, *P* = 0.006, Table 3). Further analyses using TILs showed that a combination of PD-L1 (+) and higher TIL expression counts correlated with shorter PFS, and that patients positive for both PD-L1 and TILs had the shortest PFS [5.1 vs 4.3 vs 2.7 months for both PD-L1 (−) and TIL (−) vs PD-L1 (+) or TIL (+) vs both PD-L1 (+) and TIL (+), *P* = 0.035, Fig. 3d].

**Table 3:** Prognostic Factors for Progression-free survival

	Hazard Ratio (95% CI)	P value
**Performance (0 vs 1)**	0.74 (0.39–1.44)	0.38
**Number of previous chemotherapy (1 vs 2–3)**	0.67 (0.29–1.57)	0.36
**Sex (female vs male)**	1.27 (0.65–2.49)	0.49
**Age (≤48 years vs > 48 years)**	1.69 (0.86–3.30)	0.12
**ECOG (0–1 vs 2)**	0.74 (0.39–1.44)	0.38
**Grade (I vs II-III)**	1.22 (0.43–3.45)	0.77
**Histologic subtype** **(leiomyosarcoma and synovial sarcoma** vs other subtypes^*^)	1.17 (0.49–2.06)	0.95
**Locoregional disease (yes vs no)**	1.10 (0.60–2.03)	0.75
**Liver metastases (yes vs no)**	0.75 (0.22–2.58)	0.65
**PD-L1 expression (positive vs negative)**	2.77 (1.45–5.56)	0.006
**TIL (high vs low)**	1.41 (0.83–2.37)	0.197

*other subtypes: undifferentiated pleomorphic sarcoma, angiosarcoma, myofibroblastic sarcoma, MPNST (malignant peripheral nerve sheath tumor), ASPS (Alveolar soft part sarcoma), epithelioid sarcoma, DSRCT, osteosarcoma, liposarcoma, rhabdomyosarcoma, and PECOMA

Abbreviation: Eastern Cooperative Oncology Group (ECOG), tumour infiltrating lymphocytes (TILs)

## Discussion

Using a standardised assay, in the current study we report the expression of PD-L1 in pre-treated STS tumour tissues. Furthermore, our study unravels the value of PD-L1 for predicting pazopanib efficacy. To our knowledge, this is the first study to evaluate the role of PD-L1 expression in pazopanib response in STS.

Pazopanib, an inhibitor of angiogenesis and tumour cell proliferation, has been recognized as a salvage treatment for STS [5]. The approval of this drug was based on the results of the randomised phase III PALETTE trial, which showed that pazopanib improved PFS in patients with previously treated STS compared to placebo treatment. However, despite the prolonged PFS, no significant difference in OS was observed, and our understanding of the factors mediating pazopanib sensitivity and resistance is still poor. There is an urgent need for the identification of predictive biomarkers which can be used to select subgroups of patients who will benefit from pazopanib treatment. In the PALETTE trial, it was noted that patients with leiomyosarcoma and synovial sarcoma exhibited better PFS upon pazopanib; however, a significant interaction with histological subtypes was not identified in predictive analysis. Other biological markers include post-treatment neutrophil-lymphocyte ratio, which was also suggested to be a robust predictive factor for pazopanib efficacy [7]. However, haematological parameters (such as neutrophil and lymphocyte counts) are unreliable because they can be easily influenced by other inflammatory conditions. Although a number of potential biomarkers have also been investigated, none of these are available as patient selection strategies for use in clinical practice.

Immune checkpoint inhibitors that block PD-1 and PD-L1 have exhibited remarkable efficacy with respect to the treatment of refractory solid tumours including melanoma and RCC, and substantial attention has been paid to assessing PD-L1 expression in STS. PD-L1 expression has been studied in various STS subtypes [10], and a recent meta-analysis also reported a role for PD-L1 expression in the poor prognosis of bone and STS [15]. Interestingly, immunotherapy resulted in the generation of a response in certain sarcoma subtypes, mainly UPS, liposarcoma, and angiosarcoma [8, 9]. The Cancer Genome Atlas (TCGA) analysis have described the following immune classification based on the composition of the tumor microenvironment in STS: immune-low, immune-high, and vascularized group. In this study, immune-high group (mainly UPS and liposarcoma) exhibited high expression of PD1, PDL2, and CTLA-4 [16]. Consistent with previous studies, we show that PD-L1 (+) was commonly observed in UPS, angiosarcoma, and leiomyosarcoma, and that PD-L1 (+) staining independently correlated with poor efficacy of pazopanib treatment. Furthermore, none of the synovial sarcoma tumours exhibited PD-L1 expression and this subtype exhibited a higher response to pazopanib treatment. Similarly, the synovial sarcoma subtype also showed favourable responses to pazopanib in the PALETTE trial [5]. Therefore, we propose that PD-L1 expression is associated with pazopanib resistance and that this hypothesis is worth further testing as a therapeutic strategy in future clinical trials.

A recent report described a correlation between PD-L1 expression on the tumour and the efficacy of pazopanib or sunitinib treatment in metastatic RCC. Patients whose tumours were TIL-positive and/or displayed high PD-L1 expression had poor survival outcomes in response to vascular endothelial growth factor (VEGF)-targeted agents [12]. A preclinical study showed that anti-PD-L1 therapy enhances tumour sensitivity to antiangiogenic therapy by inducing the development of endothelial venules that facilitate cytotoxic T cell activity and tumour lysis [17]. Therefore, on the basis that the VEGF receptor (VEGFR) plays a central role in immunosuppression, a strategy of combination treatments has been widely studied in RCC [18, 19]. Similarly, the combination of a VEGF inhibitor, axitinib, with pembrolizumab was shown to have promising efficacy in advanced sarcomas, particularly in patients with advanced soft-part sarcoma [20]. Taken together, the combination of pazopanib and PD-L1 blockade may be a promising future therapeutic option for STS, and the final results of an ongoing trial (A Study of Pazopanib and Durvalumab for Metastatic Soft Tissue Sarcoma; ClinicalTrials.gov Identifier: NCT03798106) may help to confirm this premise.

To clarify the exact characteristics and prognostic role of PD-L1 expression, we carefully assessed a large cohort of patients with STS who were treated with pazopanib. Furthermore, from a practical perspective, three different PD-L1 antibodies (22C3, 28–8, and SP263) showed comparable analytical performance with respect to assessing PD-L1 expression [21]. Therefore, we used a companion diagnostic marker for approved anti-PD-L1 antibodies, SP263 PD-L1, in accordance with a standard protocol (instrument platform, staining procedure, and scoring methods) to provide a practical guideline. However, because of the retrospective nature of this study, future studies should evaluate a larger, prospective cohort to address the question of whether PD-L1 expression can serve as a clinical prognostic marker for STS.

## Conclusions

Our study described a predictive role for PD-L1 expression in assessing the clinical outcomes for patients with advanced STS treated with pazopanib. This study provides important information that can guide treatment choices to better tailor the efficacy of pazopanib, thereby leading to new therapeutic strategies for sarcoma.

## Supplementary Information

**Additional file 1.**

## Data Availability

The anonymized data used and/or analyzed during the current study are available from the corresponding author on reasonable request.

## Abbreviations

STS: Soft tissue sarcoma
TPS: tumour proportion score
CTLA-4: cytotoxic T-lymphocyte–associated antigen 4
PD-L1: programmed death-ligand 1
FFPE: formalin-fixed paraffin-embedded
ECOG: Eastern Cooperative Oncology Group
FNCLCC: French Federation of Cancer Centers Sarcoma Group
TILs: tumour infiltrating lymphocytes
PFS: progression-free survival
OS: overall survival
ORR: overall response rate
CR: complete response
PR: partial response
RECIST): Response Evaluation Criteria in Solid Tumors
UPS: 1.1 undifferentiated pleomorphic sarcoma
MPNST: malignant peripheral nerve sheath tumour
ASPS: alveolar soft part sarcoma
DSRCT: desmoplastic small round cell tumors
PECOMA: perivascular epithelioid cell tumor
TCGA: The Cancer Genome Atlas
VEGF: to vascular endothelial growth factor
VEGFR: VEGF receptor
