# Supplementary material for: PD-L1 tumour expression is predictive of pazopanib response in soft tissue sarcoma
Source: BMC Cancer. 2021 Mar 31;21:336. doi: 10.1186/s12885-021-08069-z (PMC8011221; doi:10.1186/s12885-021-08069-z)
Supplement: Supplementary file 1 — Additional file 1. [file 12885_2021_8069_MOESM1_ESM.docx]

**Supplementary table 1**. Patient clinicopathological characteristics.

|  | **Clinical** | | | **Pathologic** | | **Efficacy** | | |
| --- | --- | --- | --- | --- | --- | --- | --- | --- |
| **Case No.** | **Age** | **Primary site** | **Histology** | **PD-L1 TPS** | **TIL** | **Response** | **Sum** | **PFS (day)** |
| 1 | 70s | Knee | MPNST | 100 | 10 | SD | +5% | 120 |
| 2 | 50s | Buttock | Leiomyosarcoma | 30 | 10 | SD | +3% | 167 |
| 3 | 50s | Flank | UPS | 30 | 10 | PD | +45% | 36 |
| 4 | 70s | scalp | Angiosarcoma | 30 | 5 | SD | -3% | 104 |
| 5 | 50s | scalp | Angiosarcoma | 20 | 0 | PD | +38% | 27 |
| 6 | 70s | Heart | UPS | 20 | 0 | PD | +60% | 81 |
| 7 | 40s | Arm | UPS | 10 | 30 | PD | +25% | 83 |
| 8 | 20s | Back | Leiomyosarcoma | 10 | 20 | SD | +3% | 258 |
| 9 | 70s | Rib | Solitary fibrous tumor | 10 | 0 | SD | +1% | 84 |
| 10 | 80s | Arm | UPS | 10 | 0 | SD | -3% | 159 |
| 11 | 50s | Uterus | Leiomyosarcoma | 5 | 0 | PD | +41% | 73 |
| 12 | 60s | Mediastinum | MPNST | 1 | 5 | PD | +33% | 64 |
| 13 | 20s | Brest | Angiosarcoma | 1 | 5 | PD | +10% | 70 |
